# Case Report: Gastric intramural hematoma with acute upper gastrointestinal bleeding in a child

**DOI:** 10.3389/fped.2025.1533324

**Published:** 2025-05-19

**Authors:** Ming Xiao, Jin Lu, Yu Chen, Weiwei Sun, Youcheng Sun, Yutian Li, Yang Jiang, Xingchen Lian, Zhengqiang Zhou, Xin Qi

**Affiliations:** ^1^ Department of Pediatric Surgery, Dalian Women and Children’s Medical Group, Dalian, China; ^2^ Graduate School, Dalian Medical University, Dalian, China

**Keywords:** hematoma, ulcer, gastric, interventional treatment, pediatric surgery

## Abstract

Gastric hematoma is an exceptionally rare condition in pediatric patients. It is characterized by the accumulation of blood within the gastric wall, resulting in the formation of a mass. Coagulopathy is the most common cause of gastric hematoma, although other etiologies include gastric ulcer, amyloidosis, pancreatitis, and splanchnic vascular aneurysms. However, the pathophysiology of gastric hematoma remains incompletely understood. It is presumed to be caused by ruptures of the submucosal vessels, leading to dissection of the muscular layer and the formation of a false lumen. Herein, we report the case of a 4 year-old girl who was admitted to our hospital with a large intragastric mass. She presented with hematemesis, and a large hematoma was visualized via gastroscopy. After treatment, the patient underwent a second follow-up gastroscopy, which showed that the gastric mass had subsided, and multiple gastric ulcers were found in the gastric wall. After excluding other causes, we considered that the gastric hematoma may have been caused by the ulcers. By discussing the clinical presentation and treatment options in this case, we hope to improve the understanding of pediatric gastric hematoma so that serious complications can be avoided in future cases.

## 1 Introduction

Gastric hematoma is a rare condition, particularly in children. In most cases, it is associated with trauma, coagulopathies, and peptic ulcer disease (1). Its clinical presentation is not evident, with early imaging typically suggesting a space-occupying gastric lesion. This makes gastric hematomas often challenging to diagnose and prone to causing serious complications in patients (2). Herein, we present a case where the patient suddenly developed an irregular gastric space-occupying lesion and experienced progressive upper gastrointestinal bleeding. Following ineffective conservative treatment and endoscopic hemostasis, interventional treatment was performed. We hypothesized that this patient's gastric hematoma was caused by a gastric ulcer.

## 2 Case description

**Figure 1:**
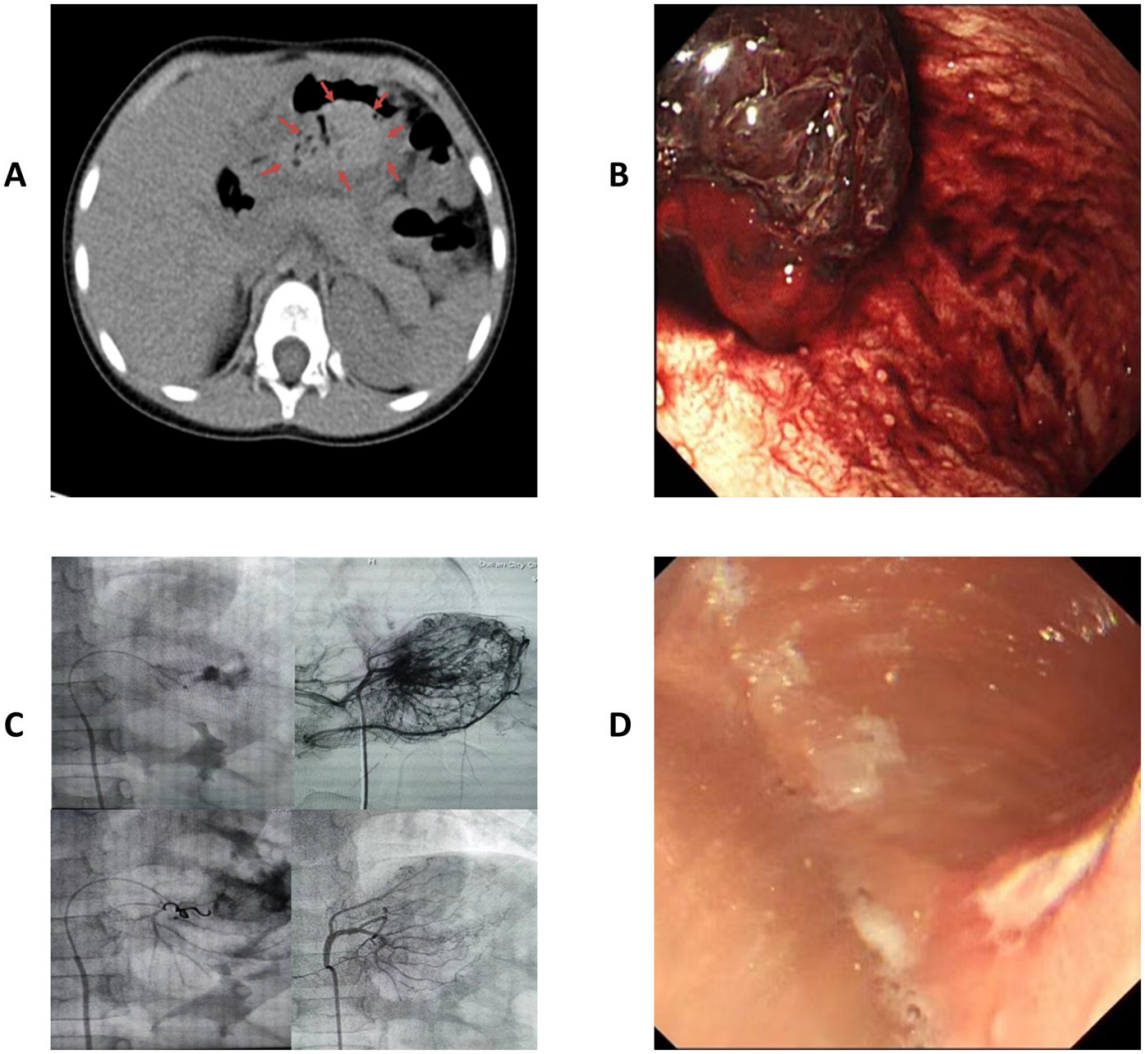
**(A)** Outpatient computed tomography showing a gastric space-occupying lesion. Arrows circumscribe the hematoma. **(B)** Gastroscopy revealed a gastric hematoma with progressive bleeding. **(C)** Interventional embolization for hemostasis. **(D)** Multiple gastric ulcers in follow-up gastroscopy 8 d postoperatively.

A previously healthy 4-year-old girl visited the emergency department with unexplained abdominal pain and vomiting lasting one day. One hour before her admission she experienced worsening symptoms and coffee ground emesis. The patient's vital signs were normal upon her admission. Emergency gastrointestinal ultrasonography and computed tomography revealed an irregular lesion in the gastric space (Figure 1A). Physical examination revealed epigastric tenderness on palpation, without rebound tenderness or muscle tension. The patient's complete blood count, coagulation function, and biochemical test results were all within normal range. Following her admission, the patient fasted and was administered symptomatic treatment that included gastrointestinal decompression, acid suppression, hemostasis, and fluid replacement. Gastrointestinal decompression continued to produce coffee ground vomitus, and blood gas analysis revealed decreased hemoglobin levels. After volume expansion and fluid replacement, a gastric wall hematoma was identified via emergency gastroscopy (Figure 1B). Gastroscopic administration of epinephrine and snake venom-derived hemocoagulase failed to achieve hemostasis. Following an urgent consultation with an interventional physician, interventional embolization was performed on the artery supplying the mass (Figure 1C), which ultimately achieved hemostasis. Postoperative examination for tumor markers did not reveal any significant abnormalities; therefore, provisional symptomatic treatment was administered. Following anti-inflammatory, acid-suppressive, and gastric mucosal-protective treatments, follow-up gastroscopy (Figure 1D) and pathological examination were performed eight days later, both of which indicated gastric ulcers. Testing for *Helicobacter pylori* yielded positive results. The patient was discharged 13 days after her admission, following adjustments to her treatment regimen. A follow-up 6 months after her discharge indicated good recovery, with no recurrence of upper gastrointestinal bleeding.

## 3 Discussion

Gastric hematomas are extremely rare and have been reported only sporadically in the literature. Their clinical presentation is often atypical, with early imaging results typically suggesting a gastric space-occupying lesion. As a result, it can be challenging to distinguish gastric hematomas from other gastric masses such as tumors, lymphomas, and abscesses (3). Owing to the reticular distribution of numerous arteries and veins in the gastric wall, as well as the large number of blood vessels in the submucosal layer, any damage to the arteries caused by external factors can lead to hematoma formation in the submucosal or muscular layer (4). In most cases, the condition's etiology is related to coagulopathy, surgery, or peptic ulcer disease (3). In the present case, a series of relevant examinations were performed upon the patient's admission to exclude coagulopathies, surgical complications, and bleeding from ruptured gastric tumors as potential causes of hematoma. Only endoscopy and pathological biopsy showed positive results after interventional hemostasis, indicating the presence of ulcerative lesions.

There are currently no definitive diagnostic or treatment methods for gastric hematomas. Endoscopic hemostasis or biopsy can exacerbate bleeding owing to uncertainty in the preliminary diagnosis (5). When a gastric hematoma is identified, the initial treatment typically requires adequate blood and volume resuscitation, in addition to correcting the coagulopathy. Most patients with the condition are hemodynamically stable, making conservative management feasible (6). This approach primarily involves blood transfusion, symptom control, correction of the underlying disease, and continuous monitoring of vital signs. If no improvement is observed, subsequent treatments may include surgery, angiography, or interventional vascular embolization (7).

In the present case, imaging performed prior to the patient's admission suggested a gastrointestinal tumor, and her clinical presentation included only abdominal pain with vomiting that was not given sufficient attention or adequately managed via symptomatic treatment. The patient developed progressive upper gastrointestinal bleeding after admission and responded poorly to conservative treatment and endoscopic hemostasis. Her hemoglobin levels also continued to decline, suggesting persistent gastric bleeding. Considering her young age, serious condition, and risk of hemorrhagic shock, percutaneous interventional embolization was chosen to achieve hemostasis, following a multidisciplinary consultation. The patient eventually recovered fully following appropriate treatment. Postoperative gastroscopy and pathological examination indicated that her gastric hematoma may have been related to gastric ulcers. The patient showed no abnormalities during the postoperative period or during subsequent follow-up visits.

Pediatric gastric hematoma is challenging to diagnose definitively during its early stages. If a diagnosis based on imaging findings is difficult and progressive bleeding occurs, gastroscopic or interventional treatment should be promptly administered to prevent serious consequences.

## 4 Patient perspective

The Dalian Women and Children's Medical Group reviewed and approved all studies involving human participants. The patient's legal guardian provided written informed consent for the publication of this case report.

## Data Availability

The raw data supporting the conclusions of this article will be made available by the authors, without undue reservation.
